# Performance evaluation of a novel RapiSafe HIV Ag/Ab combi rapid test for the detection of HIV antigen and antibodies

**DOI:** 10.3389/fcimb.2025.1646274

**Published:** 2025-10-06

**Authors:** Shuai Wei, Zhongyuan Huang, Kun Liu, Qiang Huang, Chen Lan, Qunxian Zhang, Liuling Liu, Jun Yin, Weiting Li, Yongsheng Xiang, Pengpeng Zou, Zhonggang Fang, Wenzhao Luo

**Affiliations:** ^1^ Department of Clinical Laboratory, Hechi Hospital Affiliated to Youjiang Medical University for Nationalities, Hechi, Guangxi, China; ^2^ Modern Industrial College of Biomedicine and Great Health, Youjiang Medical University for Nationalities, Baise, Guangxi, China; ^3^ Department of Infectious Diseases, Hechi Hospital Affiliated to Youjiang Medical University for Nationalities, Hechi, Guangxi, China; ^4^ Research & Development Department, Shenzhen New Industries Biomedical Engineering Co., Ltd. (Snibe), Shenzhen, China

**Keywords:** HIV diagnostics, acute HIV infection, rapid diagnostic test, RapiSafe, HIV subtypes, performance

## Abstract

**Background:**

The detection of acute HIV infection (AHI) is vital for timely diagnosis and appropriate management, thus, *in vitro* diagnostic tests that accurately identify antigen and anti-bodies separately, have a short seroconversion window period, rapid turnaround time are essential. This study aimed to evaluate the analytical and clinical performance of the RapiSafe rapid test, with a particular focus on its efficacy in detecting AHI in serum samples.

**Methods:**

Antigen sensitivity was evaluated in 1st WHO International Standard for HIV-1 p24 Antigen (NIBSC 22/230) and Reference Panel (NIBSC 16/210). Five HIV-1 seroconversion panels and one HIV-1 p24 antigen Mixed Titer Performance Panel were detected for HIV-1 p24 antigen seroconversion sensitivity evaluation. 22 treatment-naïve acute HIV-1 serum at Fiebig stage III-VI were detected with both the RapiSafe and the Determine tests. To evaluate the diagnostic sensitivity in different genotypes and specimen types of HIV-1 established infections, HIV positive serum and venous whole blood were detected. Diagnostic specificity was evaluated in clinical serum, plasma, capillary whole blood, and venous whole blood.

**Results:**

Compared with the Abbott Determine tests, the RapiSafe showed lower antigen detection limits in HIV-1 subtypes A1, C, and D; higher p24 antigen sensitivity with five more seroconversion reactive detections; and one more antigen-positive AHI serum detection. The superior antigen and antibodies sensitivity of the RapiSafe among serum, plasma, and venous whole blood did not compromise specificity (99.75-100%) among different specimen types with potentially cross-reacting substances or unrelated medical conditions.

**Conclusion:**

The exceptional performance of the RapiSafe in antigen and antibodies detection among different subtypes and specimen types makes it a valuable tool in HIV diagnosis. Its novel approach could significantly impact global HIV control by facilitating early detection and timely intervention, which are essential for the uptake of prevention, prompt HIV diagnosis, and mitigating global HIV transmission risks.

## 1 Introduction

Human immunodeficiency virus (HIV), first discovered in 1983, refers to an enveloped retrovirus with two identical single-stranded RNAs in the core of the virus particle, mainly transmitted by sexual contact, maternal-infant exposure, and percutaneous inoculation through body fluids including semen, pre-ejaculate, rectal fluids, vaginal secretions, amniotic fluid, breast milk, and blood (Barré-Sinoussi et al., 1983; Shaw and Hunter, 2012). Based on the differences in the amino acid and nucleotide characteristics, HIV could be classified into two lineages (HIV-1 and HIV-2), which evolved from two different simian immunodeficiency viruses, Central African apes and West African sooty mangabey monkeys, separately (Swinkels et al., 2024). HIV-1 is further divided into four groups (M, N, O, and P), and group M contains more than nine different subtypes, accounting for 70% of the global HIV-1 pandemic (Williams et al., 2023). In 2023, 1.3 million new HIV infections and around 630,000 deaths from acquired immunodeficiency syndrome (AIDS)-related illness were reported, among which HIV-1 accounts for more than 95% of the global infections, whereas HIV-2 only causes a few with less transmissibility and a longer asymptomatic phase (UNAIDS, 2024; Williams et al., 2023).

Despite differences between HIV-1 and HIV-2 in etiology and epidemiology, the primary host cellular receptor for the two HIV lineages is the CD4+ antigen expressed by humans’ T lymphocytes (Maartens et al., 2014). By infecting and impairing human’ T lymphocytes, HIV has devastating effects that lead to the collapse of the whole human immune system and causes profound immunodeficiency (McMichael and Rowland-Jones, 2001). With prompt diagnosis and timely antiretroviral therapy (ART), early and sufficient HIV suppression is highly likely to preserve immune response diversity, reduce inflammatory response and the size of the HIV reservoir, and lead to functional cure, especially when initiating ART during the acute HIV infection (AHI) stage (Prins et al., 2017). In addition, early and accurate HIV diagnosis is beneficial to the appropriate administration of pre-exposure prophylaxis (PrEP) and post-exposure prophylaxis (PEP), mitigating the risks of drug resistance among individuals initiating PrEP during AHI (Johnson et al., 2021).

Because of the crucial role of AHI detection in prompt HIV diagnosis and rational PrEP/PEP administration, *in vitro* diagnostic tests with high accuracy, short seroconversion window period, rapid turnaround time and improved accessibility to minimize gaps in testing service delivery are indispensable, especially to key populations including men who have sex with men, people in prisons and closed settings, people who inject drugs, sex workers, and trans and gender-diverse people. According to the latest HIV testing algorithms recommended by the World Health Organization (WHO), the HIV-1 testing strategy in health facilities with clinical laboratories can be serology assay formats involving rapid diagnostic test (RDT) and fourth-generation antigen/antibody immunoassay (4G-EIA) (Geneva: World Health Organization, 2024). And the reference standard for diagnosing AHI mainly depends on detecting both the HIV-1 p24 antigen and HIV antibodies (Bystryak and Santockyte, 2015). Compared with 4G-EIA, RDT can illustrate the presence of antigen and antibodies separately and be utilized in algorithms involving point-of-care testing, which is useful for timely diagnosis of AHI (Alexander, 2016; Livant et al., 2017).

Besides the benefits of RDT in prompt AHI detection, differentiated results presentation, and practical advantages compared with 4G-EIA, RDT would play a significant role in global HIV service and management. According to the WHO, approximately 39.9 mil-lion people were infected with HIV in 2023, and only 86% of them were aware of their HIV status, 77% were receiving ART, and 72% had suppressed viral loads (UNAIDS/WHO estimates, 2024). To achieve the UNAIDS 95-95–95 HIV care cascade targets, WHO testing strategies recommend that national algorithms use quality-assured RDTs and/or immunoassays instead of nucleic acid testing techniques, western blotting and line immunoassays for the diagnosis of individuals over 18 months of age (Geneva: World Health Organization, 2024). Specifically, improving the accessibility of RDTs and self-tests, including training community health or outreach workers to deliver HIV RDTs, is highly likely to facilitate the uptake of prevention and treatment services, ending AIDS as a public health threat by 2030 (Chamie et al., 2021; Geneva: World Health Organization, 2024; Mathews et al., 2020). Therefore, the objective of this study was to evaluate the performance of the RapiSafe HIV Ag/Ab Combi Rapid Test, our HIV RDT under development, and demonstrate the detection efficacy in serum specimens identified as AHI.

## 2 Materials and methods

### 2.1 HIV detection assays

The HIV rapid test under evaluation was the RapiSafe HIV Ag/Ab Combi Rapid Test (RapiSafe, for research use only, not commercially available), which is a sandwich colloidal gold immunochromatographic test designed and manufactured by Shenzhen New Industries Biomedical Engineering Co., Ltd. (Snibe), China. The RapiSafe utilizes mouse monoclonal antibodies against the HIV-1 p24 protein, as well as recombinantly expressed HIV-1 and HIV-2 antigens in Chinese hamster ovary (CHO) cells. These reagents are produced by Snibe and used for the qualitative detection of free HIV-1 p24 antigen (Upper Test Area) and antibodies to HIV-1 and HIV-2 (Lower Test Area). The results could be visually interpreted between 20 and 30 minutes after adding two drops (50 µL) of specimen and two drops of chase buffer (50 µL). The interpretations of all possible RapiSafe results are illustrated in **Supplementary Figure 1**.

The Abbott Determine HIV-1/2 Ag/Ab Combo rapid test (Determine), manufactured by Abbott Diagnostics Medical Co., Ltd., Japan, was performed as indicated in the package insert. All specimens were tested singlet and repeated only if invalid results were obtained (no pink control line in the Control Area of the test kit).

**Figure 1:**
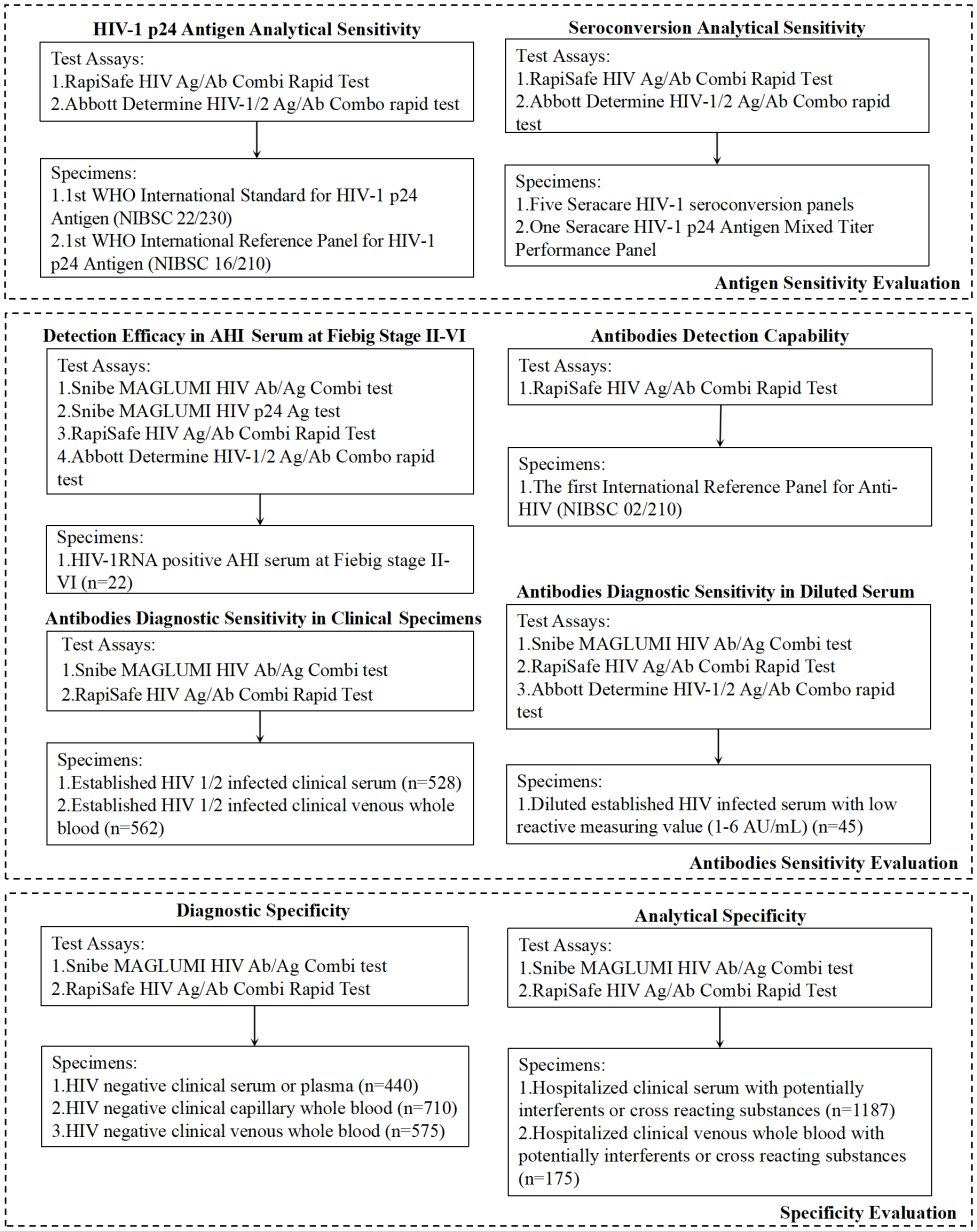
Summary of performance evaluated, test assays and specimens.

The Snibe MAGLUMI HIV Ab/Ag Combi test (MAGLUMI HIV Ab/Ag, CE and NMPA approved 4G-EIA) (Shenzhen New Industries Biomedical Engineering Co., Ltd. (Snibe), China), a two-step sandwich chemiluminescence immunoassay (Wang et al., 2024), and the Snibe MAGLUMI HIV p24 Ag test (MAGLUMI HIV p24 Ag, Shenzhen New Industries Biomedical Engineering Co., Ltd. (Snibe), China), research-use-only assay, were utilized with the MAGLUMI X8 instrument according to the manufacturer’s instructions (**Figure 1**).

### 2.2 Specimen sets

#### 2.2.1 Antigen sensitivity evaluation

HIV-1 p24 antigen analytical sensitivity was evaluated on two WHO antigen panels, including the 1st WHO International Standard for HIV-1 p24 Antigen (NIBSC 22/230) and the 1st WHO International Reference Panel for HIV-1 p24 Antigen (NIBSC 16/210). Specimens from the two panels were prepared in a series of dilutions with normal human plasma, and the relative antigen concentrations were determined based on the dilution factors.

Five HIV-1 seroconversion panels and one HIV-1 p24 antigen Mixed Titer Performance Panel, both sourced from SeraCare Life Science Inc. (Milford, MA), were tested in parallel using the RapiSafe and the Determine tests for the evaluation of HIV-1 p24 antigen seroconversion sensitivity (**Figure 1**).

#### 2.2.2 Antibodies sensitivity evaluation

22 frozen HIV-1 RNA-positive remnant serum specimens were characterized as Fie-big III-VI of AHI according to Fiebig classification, based on the reactive results of the MAGLUMI HIV Ab/Ag and MAGLUMI HIV p24 Ag (Fiebig et al., 2003). These Fiebig III-VI specimens were used to evaluate antigen and antibody detection efficacy of the RapiSafe and the Determine tests in AHI.

Serial dilutions of the 1st International Reference Panel for HIV antibodies (NIBSC code 02/210) were prepared in normal human plasma, and the corresponding concentrations of antibodies were determined based on dilution factors.

A total of 989 deidentified remnant serum (n = 427) and venous whole blood (n = 562) specimens were collected from patients with chronic HIV-1 infection during routine healthcare procedures. For the evaluation of antibodies sensitivity in different HIV groups and subtypes, 74 HIV-1 antibody-positive specimens with subtype information, as well as 27 HIV-2 antibody-positive specimens, were obtained from Biomex Sampleshop (Biomex GmbH, Heidelberg, Germany). MAGLUMI HIV Ab/Ag was applied to HIV-positive serum specimens for further classification based on reported values (AU/mL) of 6, 50, 100, 200, 300, 400, and 500. This classification approach was adopted because, according to the manufacturer’s instructions, both HIV-1 antigen and antibodies exhibit no hook effect at concentrations up to 500 AU/mL.

Owing to the number of HIV-positive clinical specimens with values below 6 AU/mL determined by MAGLUMI HIV Ab/Ag is only one and at 2.521 AU/mL, serial dilutions were prepared and determined by MAGLUMI HIV Ab/Ag. These 45 diluted serum specimens, with values ranging from 1 to 6 AU/mL, were tested in parallel by the RapiSafe and the Determine tests to investigate the diagnostic sensitivity in simulated HIV-1 anti-body-positive specimens with values ranging from 1 to 6 AU/mL (**Figure 1**).

#### 2.2.3 Specificity evaluation

A total of 1725 unselected HIV-negative specimens, including 440 serum or plasma, 710 capillary whole blood, and 575 venous whole blood specimens, were collected from hospitalized patients confirmed by the MAGLUMI HIV Ab/Ag in advance. These specimens were detected with the RapiSafe to evaluate the diagnostic specificity. All specimens were detected in a single determination with each assay. Specimens assessed with initially reactive results were retested in duplicate.

To assess the impact of potential interferents or cross-reacting substances on the analytical specificity, 1187 serum clinical specimens and 175 venous whole blood clinical specimens containing hemolysis, lipemia, bilirubin, rheumatoid factor, antinuclear anti-body, hepatitis B virus, cytomegalovirus, herpes simplex virus, syphilis, Epstein-Barr virus or toxoplasmosis, and specimens collected from pregnant women were tested with the RapiSafe (**Figure 1**).

### 2.3 Data analysis

Excel 2019 (Microsoft Inc.) was used to calculate the average value, standard deviation, coefficient of variation, proportions, and corresponding Wilson score 95% confidence intervals (CIs).

### 2.4 Ethical statement

This performance evaluation study was conducted by The People’s Hospital of Hechi (Guangxi, P. R. China) in accordance with the guidelines of the Declaration of Helsinki. The samples utilized in this study were all deidentified leftover samples with extensive informed consent. This study received ethical approval from local research ethics committee, the Research Ethical Committee of The People’s Hospital of Hechi (Ethics approval number: 2024-024-01).

## 3 Results

### 3.1 HIV-1 p24 antigen analytical sensitivity

A series of dilutions of the first International Standard (NIBSC code: 22/230) were prepared in normal human plasma and detected by three reagent pack lots. And the analytical sensitivity of the p24 antigen was determined to be no more than 2 IU/mL.

To evaluate the p24 antigen analytical sensitivity among subtypes A1, B, C, D, G, H, F1/CRF12_BF/BFrec, CRF20_BG, CRF01_AE, CRF02_AG, and group O, a two-fold series dilution of the HIV-1 p24 Antigen International Reference Panel (NIBSC code: 16/210) was performed to around 2 IU/mL. The detection results were displayed in **Table 1**. The lowest concentration still reactive with the RapiSafe antigen line was around 2 IU/mL among the 12 HIV subtypes, and the detection limits of the RapiSafe for subtypes A1, C, and D were demonstrated to be lower than those of the Determine tests.

**Table 1:** HIV-1 p24 antigen analytical sensitivity of the RapiSafe and the Determine tests using WHO 16/210 Panel and antibodies detection capability using WHO 02/210 Panel.

Panel ID	Subtype	Theoretical concentration (IU/mL)	Dilution	Rapisafe		Determine	
				HIV-1 p24 ag	HIV 1/2 ab	HIV-1 p24 ag	HIV 1/2 ab
NIBSC 16/210	A1	8.8	Neat	R	NR	R	NR
		4.4	1:2	R	NR	R	NR
		2.2	1:4	R	NR	NR	NR
	B	7.9	Neat	R	NR	R	NR
		3.9	1:2	R	NR	R	NR
		1.9	1:4	R	NR	R	NR
	B	8.4	Neat	R	NR	R	NR
		4.2	1:2	R	NR	R	NR
		2.1	1:4	R	NR	R	NR
	C	11.5	Neat	R	NR	R	NR
		3.8	1:3	R	NR	R	NR
		1.9	1:6	R	NR	NR	NR
	D	9	Neat	R	NR	R	NR
		4.5	1:2	R	NR	R	NR
		2.2	1:4	R	NR	NR	NR
	F1/CRF12_BF/Bfrec	12.7	Neat	R	NR	R	NR
		4.2	1:3	R	NR	R	NR
		2.1	1:6	R	NR	R	NR
	G	8	Neat	R	NR	R	NR
		4	1:2	R	NR	R	NR
		2	1:4	R	NR	R	NR
	CRF20_BG	9.9	Neat	R	NR	R	NR
		4.9	1:2	R	NR	R	NR
		2.4	1:4	R	NR	R	NR
	CRF01_AE	10.3	Neat	R	NR	R	NR
		5.1	1:2	R	NR	R	NR
		2.5	1:4	R	NR	R	NR
	CRF02_AG	4	Neat	R	NR	R	NR
		2	1:2	R	NR	R	NR
	H	6	Neat	R	NR	R	NR
		2	1:3	R	NR	R	NR
	group O	11.3	Neat	R	NR	R	NR
		3.7	1:3	R	NR	R	NR
		1.8	1:6	R	NR	R	NR
NIBSC 02/210	A			NR	R	NR	R
	B			NR	R	NR	R
	C			NR	R	NR	R
	E			NR	R	NR	R
	O			NR	R	NR	R
	HIV-2			NR	R	NR	R

### 3.2 Seroconversion analytical sensitivity

As shown in **Table 2**, five commercially available HIV-1 seroconversion panels were detected on both the RapiSafe and the Determine tests. And the RapiSafe detected HIV-1 p24 antigen and HIV-1 antibodies at the same bleed as the Determine tests, demonstrating that the two rapid tests have comparable seroconversion analytical sensitivity.

**Table 2:** HIV-1 p24 antigen seroconversion analytical sensitivity of the RapiSafe and the Determine tests with 5 panels.

Panel ID	Relative day of bleed	Rapisafe		Determine	
		HIV-1 p24 ag	HIV 1/2 ab	HIV-1 p24 ag	HIV 1/2 ab
PRB970	0	R	NR	R	NR
	7	R	NR	R	NR
	10	R	R	R	R
	14	NR	R	NR	R
PRB973	0	NR	NR	NR	NR
	2	NR	NR	NR	NR
	7	R	NR	R	NR
	11	R	R	R	R
PRB976	0	NR	NR	NR	NR
	2	NR	NR	NR	NR
	7	R	NR	R	NR
	9	R	NR	R	NR
PRB977	0	NR	NR	NR	NR
	2	NR	NR	NR	NR
	13	R	R	R	R
	15	R	R	R	R
PRB948	0	NR	NR	NR	NR
	18	NR	NR	NR	NR
	20	NR	NR	NR	NR
	23	R	NR	R	NR

In addition, one commercially available HIV-1 p24 Antigen Mixed Titer Performance Panel, which includes 14 HIV-1 p24 antigen-positive plasma collected from multiple individuals at varying concentrations and one non-reactive plasma for all HIV methods, was tested with the RapiSafe and the Determine tests (**Table 3**). In terms of p24 antigen sensitivity, the number of p24 antigen positive specimens detected by the RapiSafe (9/14) is five more than the Determine tests detected (4/14), but is four less than the Perkin Elmer HIV-1 p24 ELISA detected (13/14). Whilst the RapiSafe, the Determine tests and Avioq HIV-1 Microelisa were all reactive with the same 12 panel members regarding the HIV-1 antibody sensitivity. Overall, the RapiSafe demonstrated equivalent analytical sensitivity to the Determine tests in detecting HIV-1 antibody seroconversion while exhibiting slightly higher sensitivity for HIV-1 p24 antigen seroconversion.

**Table 3:** HIV-1 p24 antigen seroconversion analytical sensitivity of the RapiSafe and the Determine tests on p24 Antigen Mixed Titer Performance Panel compared with HIV-1 p24 Ag ELISA, Ab ELISA and RNA results. .

Panel ID	Panel member	Rapisafe		Determine		Perkin elmer HIV-1 p24 ELISA	Avioq HIV-1 ab microelisa ELISA	Roche HIV-1 RNA (copies/mL)
		HIV-1 p24 ag	HIV 1/2 ab	HIV-1 p24 ag	HIV 1/2 ab			
0800-0522	1	NR	R	NR	R	R	R	1.11 E + 05
	2	NR	NR	NR	NR	NR	NR	7.06 E + 02
	3	R	R	NR	R	R	R	4.91 E + 05
	4	NR	R	NR	R	R	R	5.98 E + 05
	5	R	NR	R	NR	R	NR	2.16 E + 05
	6	NR	R	NR	R	R	R	1.18 E + 06
	7	R	R	R	R	R	R	2.73 E + 04
	8	NR	R	NR	R	R	R	2.64 E + 06
	9	R	R	NR	R	R	R	6.10 E + 05
	10	R	R	NR	R	R	R	2.74 E + 06
	11	R	R	NR	R	R	R	4.15 E + 06
	12	R	R	NR	R	R	R	5.46 E + 06
	13	R	R	R	R	R	R	4.40 E + 06
	14	R	R	R	R	R	R	2.84 E + 05
	15	NR	NR	NR	NR	NR	NR	NR
Total number detected		9	12	4	12	13	12	14

### 3.3 Detection efficacy in AHI serum at Fiebig stage III-VI.

22 AHI serum specimens staged at Fiebig III-VI, according to clinical features and the reactive results of MAGLUMI HIV Ab/Ag and MAGLUMI HIV p24 Ag, were detected with both the RapiSafe and the Determine tests [18]. All of the serum specimens tested positive for antibodies using both the RapiSafe and the Determine tests. In addition, the RapiSafe detected one more p24 antigen infection (16/22) than the Determine tests (15/22), indicating the p24 antigen clinical sensitivity was the highest for MAGLUMI HIV p24 Ag (22/22), followed by the RapiSafe (16/22), and lastly the Determine tests (15/22) (**Table 4**).

**Table 4:** Detection efficacy by the RapiSafe and the Determine tests in treatment naïve AHI serum at Fiebig stage III-VI determined by HIV-1 p24 Ag EIA, Ab EIA and qualitative RNA results.

Specimen	Rapisafe		Determine		MAGLUMI HIV p24 ag	MAGLUMI HIV Ab/Ag
	HIV-1 p24 ag	HIV 1/2 ab	HIV-1 p24 ag	HIV 1/2 ab		
1	R	R	R	R	R	R
2	R	R	R	R	R	R
3	R	R	R	R	R	R
4	R	R	R	R	R	R
5	R	R	R	R	R	R
6	R	R	R	R	R	R
7	R	R	R	R	R	R
8	R	R	NR	R	R	R
9	R	R	R	R	R	R
10	R	R	R	R	R	R
11	R	R	R	R	R	R
12	R	R	R	R	R	R
13	R	R	NR	R	R	R
14	NR	R	NR	R	R	R
15	R	R	R	R	R	R
16	R	R	R	R	R	R
17	R	R	R	R	R	R
18	NR	R	NR	R	R	R
19	NR	R	NR	R	R	R
20	NR	R	NR	R	R	R
21	NR	R	R	R	R	R
22	NR	R	NR	R	R	R
Total number detected	16	22	15	22	22	22

### 3.4 Antibodies detection capability

The first International Reference Panel for Anti-HIV (NIBSC code 02/210) was used to evaluate the detection capability of HIV antibodies for HIV-2 and five different HIV-1 genetic subtypes, including subtypes A, B, C, CRF01_AE and group O. The RapiSafe detected all six anti-HIV positive specimens (**Table 1**).

### 3.5 Antibodies diagnostic sensitivity in established HIV 1/2 infected clinical specimens.

The antibodies diagnostic sensitivity of the RapiSafe was assessed by testing 1090 established HIV 1/2 infected clinical specimens in different specimen types, different genetic subtypes, and wide measuring values determined by MAGLUMI HIV Ab/Ag. **Table 5** shows that the antibodies diagnostic sensitivity of the RapiSafe was 100% in all specimen types, including serum (528/528) and venous whole blood (562/562) positive specimens from established HIV 1/2 infections. Meanwhile, the measuring values of 528 HIV-1 infected clinical serum determined by the MAGLUMI HIV Ab/Ag have covered a reactive range from 6.56 AU/mL to larger than 500 AU/mL, suggesting that the RapiSafe can detect established HIV-1 infected serum with various concentrations of HIV-1 antibodies and in concordance with the 4G-EIA assay.

**Table 5:** Antibodies diagnostic sensitivity of the RapiSafe in established HIV 1/2 infected clinical specimens compared with the MAGLUMI HIV Ab/Ag.

Specimen features	Specimen type	MAGLUMI HIV Ab/Ag (AU/mL)	Number of reactive specimens by RapiSafe	
			HIV-1 p24 ag	HIV 1/2 ab
Positive Specimens	Serum	1-6	0	1
		6-50	0	14
		50-100	0	5
		100-200	0	19
		200-300	0	18
		300-400	0	33
		400-500	0	59
		>500	0	379
	Venous Whole Blood	NA	0	562
Total			0	1090
Sensitivity	100%			
95%CI	99.65%-100.00%			

In addition, the genetic subtype information of these 1090 clinical specimens is listed in **Table 6**. By detecting the 101 specimens collected worldwide, representing a large proportion of the HIV-1 non-B subtypes and HIV-2 subtypes, the RapiSafe demonstrated high antibodies diagnostic sensitivity (100%) among a majority of HIV 1/2 subtypes and circulating recombinant forms.

**Table 6:** Genetic subtype characterization of 1090 HIV 1/2 infected clinical specimens.

Genetic subtype		Number of reactive specimens by RapiSafe	
		HIV-1 p24 ag	HIV 1/2 ab
Established HIV-1 Infected Specimens	Unspecified Subtype	0	989
	A	0	3
	A1	0	3
	B	0	4
	C	0	3
	CRF01	0	4
	CRF02	0	4
	CRF02_AG	0	1
	CRF03	0	3
	CRF06	0	2
	CRF09	0	2
	CRF11	0	4
	CRF13	0	5
	CRF14	0	3
	CRF18	0	4
	CRF22	0	2
	CRF25	0	1
	CRF37	0	2
	D	0	3
	F	0	2
	F2	0	3
	G	0	4
	H	0	3
	J	0	3
	K	0	3
	O	0	3
Established HIV-2 Infected Specimens	Unspecified Subtype	0	21
	HIV-2 Group A	0	3
	HIV-2 Group B	0	3
Total		0	1090
Sensitivity	100%		
95%CI	99.65%-100.00%		

### 3.6 Antibodies diagnostic sensitivity in diluted serum with low reactive HIV measuring values.

Because the established HIV 1/2 infected clinical specimens collected in routine healthcare processes described in the former 3.5 part failed to include clinical specimens with measuring values from 1 to 6 AU/mL determined by the MAGLUMI HIV Ab/Ag, serial dilutions of the established HIV infected clinical serum were prepared with normal human serum and tested in parallel by the MAGLUMI HIV Ab/Ag, the Determine and the RapiSafe tests to evaluate the antibodies diagnostic sensitivity in diluted serum with HIV measuring values ranging from 1 to 6 AU/mL. Results presented in **Supplementary Table S1** demonstrated that the antibodies diagnostic sensitivity of the RapiSafe (57.78%) is slightly higher than that of the Determine tests (51.11%) in simulated serum specimens, with values ranging from 1 to 6 AU/mL determined by the MAGLUMI HIV Ab/Ag.

### 3.7 Diagnostic specificity

Diagnostic specificity was evaluated in HIV-negative clinical serum/plasma (n = 440), capillary whole blood (n = 710), and venous whole blood (n = 575) specimens. All these specimens (n = 1725) were collected from hospitalized patients who were confirmed to be HIV negative on admission to hospital, and detected to be HIV unreactive by the MAGLUMI HIV Ab/Ag. Of the total 1725 HIV-negative specimens, the RapiSafe showed 100% (95% CI: 99.78%-100%) concordance with the MAGLUMI HIV Ab/Ag across all three different types, and both the RapiSafe and the MAGLUMI HIV Ab/Ag demonstrated 100% diagnostic specificity (**Supplementary Table S2**).

### 3.8 Analytical specificity

1187 clinical serum and 175 clinical venous whole blood specimens collected in routine healthcare settings with potentially interfering or cross-reacting substances were detected to evaluate the analytical specificity of the RapiSafe. There were three clinical serum collected from pregnant women tested to be false reactive, including two false-positive results for the p24 antigen and one false-positive result for antibody (**Table 7**). The remaining 1184 serum, including 460 HIV-negative serum collected from pregnant women and 175 HIV-negative venous whole blood, all gave non-reactive detection results (**Table 7**). The analytical specificity of the RapiSafe proves to be 99.75% in serum and 100% in venous whole blood with potentially interfering or cross-reacting substances, respectively.

**Table 7:** Analytical specificity of the RapiSafe in clinical specimens with potentially interfering or cross-reacting substances.

Specimen features	Serum		Venous whole blood	
	Reactive	Non-reactive	Reactive	Non-reactive
Hemolysis	0	2	N/A	N/A
Lipemia	0	73	0	10
Bilirubin	0	91	0	21
Rheumatoid factor	0	54	0	6
Antinuclear antibody	0	51	0	1
Hepatitis B Virus	0	79	0	87
Cytomegalovirus	0	86	0	6
Herpes Simplex Virus	0	85	0	6
Syphilis	0	86	0	1
Epstein Barr Virus	0	100	N/A	N/A
Toxoplasmosis	0	17	N/A	N/A
Pregnant women	3	460	0	37
Total	3	1184	0	175
Analytical specificity	99.75%		100.00%	
95%CI	99.26%-99.91%		97.85%-100.00%	

## 4 Discussion

In this study, we have demonstrated that RapiSafe exhibits exceptional sensitivity and specificity for antigens and antibodies across various specimen types. Notably, it shows superior p24 antigen sensitivity in seroconversion panels and in AHI serum at Fiebig stages III-VI. Furthermore, RapiSafe achieves lower antigen detection limits for HIV-1 subtypes A1, C, and D compared to the Determine tests.

The 2019 consolidated guidelines on HIV testing services highlighted that the increased accessibility of rapid diagnostic tests has improved the delivery of HIV testing services in both the routine health facility testing and community-based outreach within low- and middle-income countries (World Health Organization, 2020). These tests offer same-day diagnosis more swiftly than western blotting, thereby reducing the percentage of loss to follow-up. Additionally, they are cost-effective and provide reliable results (World Health Organization, 2020). Several studies have demonstrated that the Determine test has higher antigen sensitivity in AHI and detects HIV infection earlier than the third-generation rapid test (Livant et al., 2017; Stafylis and Klausner, 2017). In this study, the RapiSafe and the Determine tests concurrently detected five commercially available HIV-1 seroconversion panels, one HIV-1 p24 Antigen Mixed Titer Performance Panel, and 22 clinical specimens with AHI at Fiebig stage III-VI. The results showed that the RapiSafe detected five more HIV-1 p24 antigen-positive plasma and one more clinical serum specimen than the Determine tests. This indicates that the RapiSafe is likely to offer reliable detection results in AHI, with higher antigen sensitivity than the Determine tests.

The latest phylogenetic analysis of the global distribution of HIV-1 subtypes reveals that the top three most prevalent HIV-1 subtypes are CRF/URFs (29%), subtype C (23%) and subtype A (17%) (Williams et al., 2023). Geographically, CRFs are prevalent in Asia and West Africa, subtype C is most common in Southern Africa and India, and subtype A is primarily found in East Africa, Russia, and parts of Eastern Europe (Bbosa et al., 2019). To evaluate the analytical sensitivity and detection limits of the p24 antigen among different HIV-1 subtypes, the HIV-1 p24 Antigen International Reference Panel (NIBSC code: 16/210) was diluted to approximately 2 IU/mL. The RapiSafe and Determine tests were then used to assess 12 specimens representing various HIV-1 subtypes, including A1, B, C, D, F1/CRF12_BF/BFrec, G, CRF20_BG, CRF01_AE, CRF02_AG, H, and O. **Table 1** shows that both tests were reactive with all neat specimens, indicating their antigen sensitivity among different HIV-1 subtypes. However, the Determine test showed a higher limit of detection (>2 IU/mL) for subtypes A1, C, and D, while the RapiSafe maintained detection limits around 2 IU/mL for all these subtypes. In conclusion, the RapiSafe meets the global antigen detection requirement with lower detection limits (around 2 IU/mL) and p24 antigen analytical sensitivity across various HIV-1 subtypes compared to the Determine tests, particularly in subtypes A1, C, and D.

The RapiSafe demonstrated superior antigen and antibody sensitivity across serum, plasma, and venous whole blood specimen types without compromising diagnostic specificity, which remained at 100% for serum, plasma, capillary whole blood, and venous whole blood (**Supplementary Table S2**). However, during the evaluation of analytical specificity, the RapiSafe produced two false-positive antigen results and one false-positive antibody result in clinical serum collected from pregnant women. These same samples were tested using the Determine assay, which yielded negative results. Previous research has assessed the specificity of the Determine assay and three other FDA-approved third-generation HIV antibody rapid tests. The Determine assay showed a significantly lower specificity (1071/1077), with two false-positive antibody results and four false-positive antigen results. This suggests that fourth-generation HIV antigen and antibody diagnostic assays may occasionally produce higher false-positive results compared to third-generation HIV antibody diagnostic assays (Chavez et al., 2020). False-positive HIV serology may arise from cross-reactive antibodies to HIV 1/2 generated by polyclonal B-cell stimulation in patients with various other infections and inflammatory conditions. Less frequently reported false-positive antigen testing may be attributed to assay dysfunction or malfunction (Wormser and Schneider, 2024). Consequently, multicenter field evaluations, head-to-head comparisons with other rapid tests, and assessments of usability qualification by external laboratories and third-party organizations are necessary to more thoroughly evaluate the clinical performance of the RapiSafe, with a particular focus on vulnerable populations, including pregnant women and children.

This study has several limitations that should be considered. Firstly, the antigen and antibodies sensitivity in HIV-positive capillary whole blood specimens were not assessed in our clinical performance evaluation. This was primarily due to the hospital’s routine exclusion of this specimen type from HIV management procedures. Capillary whole blood is widely used in community-based testing and laboratory-based testing (Tang et al., 2017), and WHO strongly recommends that capillary whole blood specimen collection and point-of-care testing with nonlaboratory personnel should be implemented to increase access of HIV testing services in community-based and community outreach settings (World Health Organization, 2021). Further multicenter prospective studies are required to better characterize the clinical performance of the Rapisafe in capillary whole blood. Additionally, while antibody detection capacity was evaluated in HIV-2 and five different HIV-1 subtypes (NIBSC 02/210), the detection limits for these subtypes were not determined through serial dilutions of the reference panel, as the specimen volume was limited. Furthermore, although the RapiSafe demonstrated exceptional HIV-1 p24 antigen analytical and clinical sensitivity compared to the Determine tests, its detection capacity for HIV p24 antigen in serum or whole blood spiked with biotin has not been evaluated. The impact of biotin on the performance of the RapiSafe, particularly its HIV-1 p24 antigen sensitivity, is of critical importance. Owing to its small molecular weight and high affinity to streptavidin, biotin, a water-soluble B-complex vitamin naturally present in food and available as a dietary supplement, has been extensively used to immobilize biotinylated proteins together with immobilized streptavidin to capture and detect an analyte in many *in vitro* diagnostic immunoassays using the streptavidin-biotin immobilization system (Bayart et al., 2020; Haleyur Giri Setty et al., 2020). Research indicates that excess free biotin in a blood sample is the leading cause of false negative results among HIV 4G-EIA and HIV RDTs that rely on the streptavidin-biotin immobilization system (Haleyur Giri Setty et al., 2020; Bayart et al., 2020; Ince and Sezgintürk, 2022). This interference occurs because free biotin competes with biotinylated antibody-antigen complexes, disrupting the intended binding interactions. According to the instruction for use of the Determine tests and published literature, the monoclonal biotinylated antibody for p24 is applied in the Determine test kit for the detection of p24 antigen, and the Determine may produce false negative results for the p24 antigen in samples from AHI patients who are taking biotin (Haleyur Giri Setty et al., 2020). Even though the Rapisafe uses mouse monoclonal anti-p24 antibody to detect p24 antigen and biotin is not employed as a reagent component, further study is necessary to evaluate potential biotin interference on the analytical and clinical sensitivity of the Rapisafe. In 2024, 87% of all individuals living with HIV were aware of their status, while the proportion of children living with HIV who knew their status was 63% (UNAIDS, 2024). To meet the first 95–95–95 target and eliminate AIDS as a public health threat by 2030, the HIV status of an additional 3.2 million individuals living with HIV must be determined (UNAIDS, 2024). and high-performance fourth-generation HIV RDTs would play a crucial role in expanding access to HIV testing services in community-based and community outreach as well as primary care settings, thereby enabling timely diagnosis and further minimizing the risk of misdiagnosis (World Health Organization, 2024). The RapiSafe delivers accurate detection results for acute and chronic HIV infections within 20 minutes, without necessitating professional staffing, sophisticated laboratory infrastructure, or constant electrical supply. These features are beneficial to facilitating access and uptake of HIV testing, timely initiating ART, and reducing loss to follow-up in low-resource settings. They could also ensure appropriate allocation of healthcare resources, prevent unnecessary treatment, and further reduce HIV transmission rates, thereby protecting patients and communities in a cost-effective manner.

In conclusion, the RapiSafe has been demonstrated to be a valuable rapid diagnostic tool that offers enhanced seroconversion sensitivity in acute HIV infection (AHI). It exhibits robust antigen analytical sensitivity across various genetic subtypes and maintains high antigen and antibody sensitivity in serum and venous whole blood specimen types. Additionally, the RapiSafe achieves high specificity in serum, plasma, capillary whole blood, and venous whole blood samples. The RapiSafe could significantly contribute to the global efforts to enhance HIV prevention, facilitate timely HIV diagnosis, and reduce HIV transmission risks. By providing a rapid, and accurate diagnostic tool, the RapiSafe has the potential to improve HIV testing uptake and early detection, ultimately aiding in the mitigation of the HIV epidemic on a large scale.

## Data Availability

The original contributions presented in the study are included in the article/**Supplementary Material**. Further inquiries can be directed to the corresponding author.
