# Supplementary material for: Performance evaluation of a novel RapiSafe HIV Ag/Ab combi rapid test for the detection of HIV antigen and antibodies
Source: Front Cell Infect Microbiol. 2025 Oct 6;15:1646274. doi: 10.3389/fcimb.2025.1646274 (PMC12535964; doi:10.3389/fcimb.2025.1646274)
Supplement: Supplementary file 1 [file DataSheet1.pdf]

Supplementary Material

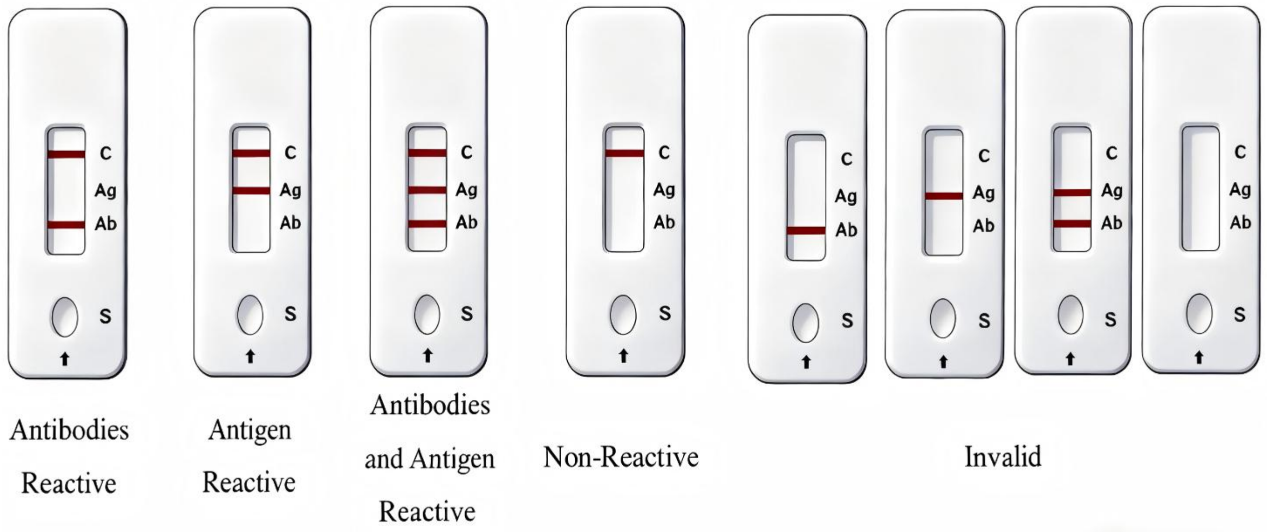

Supplementary Figure 1. Interpretations of all possible results from the RapiSafe HIV Ag/Ab Combi Rapid Test. Antibody Reactive result: Two red bands appear. One is located in the testing area (Ab) and the other is located in the quality control area (C). The strip color of the testing area (Ab) can be dark or light, and all are reactive results. Antigen Reactive result: Two red bands appear. One is in the testing area (Ag) and the other is in the quality control area (C). The color of Ag bands can be dark or light, and the results are all reactive. Antibody Reactive and Antigen Reactive: Three red bands appear. One is in the testing area (Ab), one is in the testing area (Ag), and the third is in the quality control area (C). In the testing area (Ab, Ag), the band color can be dark or light, and the results are reactive. Negative result: A colored line only appears in the quality control area (C) line region, but the testing area (Ab, Ag) line region is not visible. Invalid result: If no quality control area (C) line is visible, no matter whether the testing area (Ab, Ag) line is visible or not, the test result should be considered invalid.

Table S1. Antibodies diagnostic sensitivity of the RapiSafe and the Determine in 45 diluted serum with HIV measuring values ranging from 1 to 6 AU/mL.

Number of Diluted Serum (1-6 AU/mL)	Number of Reactive Specimens by RapiSafe		Number of Reactive Specimens by Determine	
	HIV-1 p24 Ag	HIV 1/2 Ab	HIV-1 p24 Ag	HIV 1/2 Ab
45	0	26	0	23
Sensitivity	57.78%		51.11%	
95%CI	43.30%-71.03%		37.00%-65.04%	

Table S2. Diagnostic specificity of the RapiSafe in HIV-negative clinical specimens compared with the MAGLUMI HIV Ab/Ag.

Specimen Type	RapiSafe	MAGLUMI HIV Ab/Ag
Serum/Plasma (n = 440)	100% (99.13%-100.00%)	100% (99.13%-100.00%)
Capillary Whole Blood (n = 710)	100% (99.46%-100.00%)	100% (99.46%-100.00%)
Venous Whole Blood (n = 575)	100% (99.34%-100.00%)	100% (99.34%-100.00%)
Total (n = 1725)	100% (99.78%-100.00%)	100% (99.78%-100.00%)
